# New software

**DOI:** 10.1155/S1463924601000116

**Published:** 2001


### New software

### Alias Limited Release I-Sketch Version 2.2

Alias Limited, marketer of piping isometric software, today announced the availability of I-Sketch Version 2.2. Already recognized as the best in its class, the new features and enhancements puts I-Sketch even further ahead of the field in terms of its intuitive features, ease of use, and increased productivity.

Available either as a stand-alone version, I-Sketch Classic, or as part of a design integration suite which has the ability to integrate with a 3D Plant Design System, I-Sketch is a complete package that simplifies the task of creating isometric drawings by non-CAD trained engineering personnel.

The new version incorporates a number of features for improving efficiency further and increasing productivity. Developments include additional pipe editing facilities to enable users to pick up and move a section of pipe and all its fittings and a 'break a pipe' feature to allow insertion of new components or to change a bore. Pipes can now be easily extended to connect with a bend or elbow.

The pipe import facility has been greatly improved and sections of pipe can be inserted into an existing file and placed where required. Batch drawing production is available to increase efficiency and productivity as an optional extra to I-Sketch Classic and is included as standard in the I-Sketch Enterprise package.

I-Sketch produces industry standard isometrics in minutes; implementation requires minimal time and effort and it is easy to learn. It produces multiple output formats, is highly customizable and supports a wide range of reporting functions.

### More on Alias

Alias Limited is based in the north-west of England and specializes in the development of software for the process industries. The core competence of the company is automating labour intensive operations in plant design with a particular focus on pipes.

Alias leads the world in the automatic isometric generation of piping with their flagship product, ISOGEN, which is available from many of the world's leading plant design vendors, including Intergraph, Rebis, Bentley, Tribon Solutions AB, Aquaconsult, CEA International, Profox, COADE and PRO-CAD. Alias's product portfolio includes SPOOLGEN, for the production and management of fabrication spool isometrics, and I-Sketch, the most recent addition, for the automatic production of piping isometrics from simple sketches.

The company was formed in 1991. Alias software products are available world-wide via a comprehensive network of resellers.

*More information about Alias is available at http://www.alias.ltd.uk*

*Journal of Automated Methods & Management in Chemistry* ISSN 1463–9246 print/ISSN 1464–5068 online © 2001 Taylor & Francis Ltd
http://www.tandf.co.uk/journals
DOI: 10.1080/146392401159050

